# Is Protein Phosphatase Inhibition Responsible for the Toxic Effects of Okadaic Acid in Animals?

**DOI:** 10.3390/toxins5020267

**Published:** 2013-02-04

**Authors:** Rex Munday

**Affiliations:** AgResearch Ltd, Ruakura Research Centre, Private Bag 3123, Hamilton, New Zealand; E-Mail: rex.munday@agresearch.co.nz; Tel.:+64-7-838-5138; Fax: +64-7-838-5012

**Keywords:** okadaic acid toxicity, protein phosphatase inhibition

## Abstract

Okadaic acid (OA) and its derivatives, which are produced by dinoflagellates of the genera *Prorocentrum* and *Dinophysis*, are responsible for diarrhetic shellfish poisoning in humans. In laboratory animals, these toxins cause epithelial damage and fluid accumulation in the gastrointestinal tract, and at high doses, they cause death. These substances have also been shown to be tumour promoters, and when injected into the brains of rodents, OA induces neuronal damage reminiscent of that seen in Alzheimer’s disease. OA and certain of its derivatives are potent inhibitors of protein phosphatases, which play many roles in cellular metabolism. In 1990, it was suggested that inhibition of these enzymes was responsible for the diarrhetic effect of these toxins. It is now repeatedly stated in the literature that protein phosphatase inhibition is not only responsible for the intestinal effects of OA and derivatives, but also for their acute toxic effects, their tumour promoting activity and their neuronal toxicity. In the present review, the evidence for the involvement of protein phosphatase inhibition in the induction of the toxic effects of OA and its derivatives is examined, with the conclusion that the mechanism of toxicity of these substances requires re-evaluation.

## 1. Introduction

**Figure 1:**
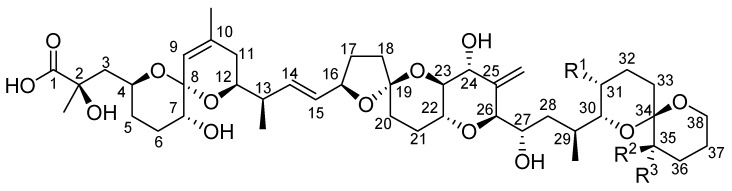
General structure of okadaic acid and derivatives.

Okadaic acid (OA, Figure 1, R_1_ = CH_3_, R_2_ = H, R_3_ = H) is produced by dinoflagellates of the genera *Prorocentrum* and *Dinophysis*, which are of worldwide distribution [1]. These organisms also produce the isomeric compounds dinophysistoxin 2 (DTX-2, Figure 1, R_1_ = H, R_2_ = H, R_3_ = CH_3_) and 19-*epi*-okadaic acid, together with the methylated derivative, dinophysistoxin 1 (DTX-1, Figure 1, R_1_ = CH_3_, R_2_ = CH_3_, R_3_ = H). Many esters formed from OA and the dinophysistoxins by conjugation of the terminal carboxylic acid group with poly-hydroxylated, sulphated or unsaturated alcohols have also been isolated from these organisms [2]. When ingested by shellfish, a proportion of the toxins present in the dinoflagellates are acylated at the C-7 hydroxyl group with long-chain fatty acids, forming derivatives collectively known as DTX-3 [3].

Shellfish contaminated with OA and its derivatives are responsible for Diarrhetic Shellfish Poisoning (DSP) in humans. DSP was first reported in Japan in the late 1970s [4], but since that time, it has been recorded in many other parts of the world, including Europe, Scandinavia, North and South America and New Zealand [5,6]. The predominant symptoms associated with DSP are nausea, vomiting, diarrhoea and abdominal pain, observed soon after ingestion of contaminated shellfish. Symptoms generally resolve within 2–3 days, and no deaths associated with DSP have been reported.

## 2. Toxicity of Okadaic Acid and Derivatives to Experimental Animals

### 2.1. Acute Toxicity of Okadaic Acid and Derivatives

OA itself is highly toxic to mice by intraperitoneal injection, with LD_50_ values between 192 and 225 µg/kg being reported [7,8,9,10]. Although no LD_50_ data on DTX-1 are available, it would appear that this compound, with a reported minimum lethal dose of 160 µg/kg [11], is of similar toxicity to OA. DTX-2, DTX-3 and DTX-4 are somewhat less acutely toxic, with LD_50_ values between 352 and 600 µg/kg [7,12,13]. Acylation of the 7-hydroxyl group with a saturated fatty acid, to form 7-*O*-palmitoyl-OA or a di-unsaturated fatty acid, giving 7-*O*-linoleoyl-OA, greatly decreases toxicity, and these compounds are approximately 20-times less toxic than OA. In contrast, the polyunsaturated 7-*O*-docosahexaenoyl-OA was reported to be 10-times more toxic than the saturated or di-unsaturated esters, with a minimum lethal dose of 550 µg/kg [14], indicating that this substance is of similar toxicity to DTX-2, DTX-3 and DTX-4 and approximately half as toxic as OA. 

OA is less toxic by oral administration than by intraperitoneal injection, although the data that are available are inconsistent. The median lethal dose of OA by gavage was reported as 400 µg/kg [15] and 880 µg/kg [16], while Tubaro *et al.* [9] observed no deaths at 1,000 µg/kg. Even within the same laboratory, wide variations in estimates of the median lethal dose occur. Le Hégarat [17] found 100% mortality in mice dosed with OA at 300 µg/kg in one experiment, but none at 610 µg/kg in a subsequent one. The reason or reasons for these disparate results are unknown, but it would appear that OA is between 2- and 5-fold less toxic by oral administration than by injection. Few data are available on the OA derivatives, but it would appear that DTX-1 is only slightly less toxic when administered orally than when given by intraperitoneal injection [18,19].

Severe damage to the epithelium of villi in the duodenum and upper jejunum, with separation and desquamation of epithelial cells from the lamina propria, was seen after intraperitoneal injection of OA in mice, although little effect was seen in the crypts. Pronounced oedema of the lamina propria was observed, attributed to increased permeability of vessels of the intestinal villi [20]. Erosion of the intestinal epithelium was recorded in animals injected with OA and DTX-1, while little effect on the gastrointestinal tract was observed in animals receiving the same dose of DTX-3 [18,21]. The toxic changes induced in the small intestine of mice by intraperitoneal injection of OA and its derivatives were also observed after oral administration of these substances, and some epithelial damage was also observed in the caecum and large intestine of these animals [9,15,18,21]. Oral administration of OA also caused oedema and mucosal erosion in the stomach of mice, accompanied by acute inflammatory changes in the submucosa [9,15,22]. Oral administration of OA to rats induced changes in the gastrointestinal tract similar to those seen in mice [23].

The cause of death following administration of lethal doses of OA is presently unclear. After intraperitoneal injection, mice showed hypothermia and muscular paralysis (particularly in the hind legs) and respiratory paralysis [8], and the latter may have been responsible for the death of the animals. In contrast, Ito and Terao [18] attributed death after intraperitoneal injection to hypovolaemic shock following haemorrhage and congestion in the liver. Congestion of blood in the liver, associated with dissociation of biliary canalicular actin sheaths, was also observed in rats following intravenous administration of OA [23]. Other authors, however, have reported relatively minor hepatic effects (isolated necrosis, lipidosis or vacuolation of hepatocytes) after injection of OA [9]. No liver damage was observed in mice or rats dosed orally with OA at lethal doses [15,21,23].

### 2.2. Diarrhoeagenicity of Okadaic Acid

After intraperitoneal injection of OA in mice, distension of the duodenum and upper jejunum was observed, associated with fluid accumulation in the lumen [9,20]. In a repeated-dose experiment with OA, five mice were dosed by gavage at a dose of 1,000 µg/kg/day for seven days. Diarrhoea was observed in all of the mice. In three animals, this ceased within a few hours, but in two of the mice, the diarrhoea was profuse and persistent, and these animals died after the fifth dose of the test compound. The surviving mice were killed on the eighth day of the experiment. At necropsy, the small intestines of the animals were observed to be full of fluid [24].

### 2.3. Toxicity of Okadaic Acid and Derivatives through Dermal Application

OA and DTX-1 have been shown to cause severe irritation when applied to mouse skin [25,26].

### 2.4. Tumour Promotion by Okadaic Acid and Derivatives

Repeated application of OA or DTX-1 to mouse skin was shown to promote tumour formation following initiation with 7,12-dimethylbenz[*a*]anthracene (DMBA) [25,26,27]. OA also acted as a tumour promoter in the rat glandular stomach after initiation with *N*-methyl-*N’*-nitro-*N*-nitrosoguanidine (MNNG) [28].

### 2.5. Neurotoxicity of Okadaic Acid after Intra-Cerebral Injection

Injection of OA into the brain of rats causes neuronal cell death [29,30,31,32,33,34,35] and memory impairment [35,36,37,38,39]. In contrast, DTX-1 caused no neuronal death when injected into the brain at a dose equivalent to that at which OA caused severe neurodegeneration [31].

It has been reported that the neuronal damage induced by OA is accompanied by hyperphosphorylation of tau protein, increased formation of neurofibrillary tangles and deposits of β-amyloid [35,40,41,42,43], although other authors have been unable to replicate such effects [29,34].

## 3. Inhibition of Protein Phosphatases by Okadaic Acid and Derivatives

In 1988, Bialojan and Takai [44] reported that OA inhibited certain protein phosphatases *in vitro* at nanomolar concentrations. The inhibitory action of OA is greater against PP2A than against PP1 [45]. It is also a potent inhibitor of PP4 and PP5 [46]. This compound has proved valuable in the study of the functions of protein phosphatases in cells *in vitro* [46,47].

DTX-1 is a somewhat more potent inhibitor of protein phosphatases than OA [45,48,49,50], while DTX-2 is half as active [7]. DTX-4 is much less effective (~500-times less active than OA) [51], while 7-*O*-palmitoyl-OA is a very weak inhibitor, more than 3,000-times less active than OA [45]. 7-*O*-Docosahexaenoyl-OA is a little more effective than the palmitoyl derivative, showing 64% inhibition at a concentration of 1 mg/ml, compared to 40% for the palmitoyl derivative at the same concentration, but it is clearly still a very weak inhibitor [50].

The ability of OA and derivatives to inhibit protein phosphatases has been utilised in the development of sensitive assays for DSPs in shellfish [52,53,54,55].

## 4. Involvement of Protein Phosphatase Inhibition in the Toxic Effects of Okadaic Acid

In 1990, Cohen *et al.* [56] published a review of the use of OA in the study of the biological processes involving protein phosphatases and stated that OA “probably” causes diarrhoea by stimulating the phosphorylation of proteins controlling sodium secretion by intestinal cells, although no evidence for this suggestion was given. This statement has been repeated many times in the literature, often with omission of the word “probably”, and, despite publications questioning the association between protein phosphatase inhibition and toxicity [57,58], it is often implied or stated, without supporting evidence, that inhibition of protein phosphatases is responsible not only for the diarrhoeagenicity of OA, but also for its acute toxic effects, its tumour-promoting activity and its neurotoxicity [2,5,15,23,24,31,48,50,52,53]. As discussed below, however, there is conflicting evidence for the validity of this suggestion, and the possible role of protein phosphatase inhibition in the toxic effects of OA and its derivatives requires re-evaluation.

Support for a causal relationship between protein phosphatase inhibition and toxicity would be gained from a number of pieces of evidence:

1. Demonstration of protein phosphatase inhibition *in vivo*, at the sites at which toxicity has been observed.
2. Proportionality between the efficacy of OA derivatives in inhibition of protein phosphatases and the severity of the toxic effects that they induce.
3. The induction of toxic effects similar to those observed with OA by other inhibitors of protein phosphatases.
4. A defined pathway from protein phosphatase inhibition to the observed toxic effects.

### 4.1. Protein Phosphatase Inhibition and Acute Toxicity of Okadaic Acid

Comparisons between the relative acute toxicities of OA and its derivatives, as measured by intraperitoneal injection in mice, and the relative activities of these substances in inhibiting PP2A and PP1 are shown in Table 1.

**Table 1:** Relative toxicities of OA and derivatives to mice by intraperitoneal injection and relative inhibitory activities toward protein phosphatases.

Compound	Relative toxicity to mice (i.p. injection)	Relative inhibition of PP2A	Relative inhibition of PP1
OA	1.0	1.0	1.0
DTX-1	1.0 [11]	1.6–2.4 [45,48,49]	0.4–0.9 [45,48]
DTX-2	0.6 [7]	0.5 [7]	-
DTX-4	0.3 [13]	0.002 [51]	-
7-*O*-palmitoyl-OA	0.05 [14]	<0.0003 [45]	<0.00005 [45]
7-*O*-docosahexaenoyl-OA	0.5 [14]	<0.0003 [45,50]	-

The equivalent relative potency of OA and DTX-2 with regard to toxicity and enzyme inhibition is consistent with the involvement of protein phosphatases in the acute toxicity of these substances. Similarly, the relatively non-toxic 7-*O*-palmitoyl derivative is a weak inhibitor of the protein phosphatases. However, a rather higher acute toxicity of DTX-1 might have been expected in view of its relatively high potency against PP2A, and while DTX-4 is ~500-times less effective as a protein phosphatase inhibitor than OA, it is only three-times less toxic to mice when injected intraperitoneally. Similarly, 7-*O*-docosahexaenoyl-OA is a very weak inhibitor of protein phosphatases, yet it is highly toxic to mice.

It could be argued that the relatively high acute toxicities of DTX-4 and 7-*O*-docosahexaenoyl-OA reflect saponification of the ester function within the peritoneal cavity, yielding OA. While esters of OA are known to be hydrolysed under acid or alkaline conditions or through the action of esterases or lipases within the intestine [59], saponification of injected esters is unlikely under the neutral conditions pertaining in the peritoneum and in the absence of the hydrolytic enzymes. Furthermore, if the esters are converted to OA in the peritoneum, it would be expected that 7-*O*-palmitoyl-OA would be of similar toxicity to 7-*O*-docosahexaenoyl-OA, since it is difficult to see why the toxic unsaturated ester should be saponified more readily than the relatively non-toxic saturated ester.

The possibility that OA-induced death is due to hypovolaemic shock following haemorrhage in the liver [18] is of interest, since other compounds known to inhibit protein phosphatases, including microcystin-LR [60,61,62], cantharidin and cantharidic acid [63,64] and acetaminophen [65,66], produce similar hepatic damage. However, such hepatotoxicity is not necessarily attributable to protein phosphatase inhibition. Inhibition of protein phosphatases has not been demonstrated in the livers of animals dosed with OA or its derivatives, and similar effects in the liver are induced by phalloidin [67] and saponin [68], which are not known to inhibit these enzymes. Furthermore, it must also be noted that the hepatic haemorrhage has not been seen in all studies involving injection of OA, and it is not seen after oral administration, even though OA causes death by this route and is taken up into the liver after administration by gavage [15].

The results of other studies [8] suggest that death may be attributable to respiratory paralysis, and OA has been shown to inhibit the twitch response in the mouse phrenic nerve hemidiaphragm preparation [69]. It may be that OA causes death through inhibition of neuromuscular transmission, as has been demonstrated with many other natural toxins [70]. In this context, the report of Valdiglesias *et al.* [71] that OA has significant effects on genes concerned with synaptic transmission in SHSY5Y neuroblastoma cells is of interest.

More work on the acute toxicity of OA is required, particularly with regard to the mechanism of death and the association between toxicity and protein phosphatase inhibition *in vivo*. At present, it must be concluded that the suggestion that OA-induced death is due to protein phosphatase inhibition is not proven.

### 4.2. Protein Phosphatase Inhibition and Diarrhoeagenicity of Okadaic Acid

Protein phosphatase activity was significantly decreased in homogenates of the intestines of mice 3 and 6 hours after oral administration of OA at 750 µg/kg [72], an observation that would satisfy the first criterion for an association between enzyme inhibition and toxicity. However, it should be noted that sloughing of intestinal villi was also observed at these time intervals, and it is possible that cellular loss could have contributed to the observed change in protein phosphatase activity.

The often-cited suggestion that OA causes diarrhoea by mediating hyperphosphorylation of proteins controlling sodium secretion [56] was disproved by subsequent studies by Tripuraneni *et al.* [73], who showed that OA has no significant effect on ion currents in intestinal cell monolayers. It does, however, increase paracellular permeability in such monolayers *in vitro* [73,74] and in the rat colon *in vivo* [75], and it was suggested that such an effect would lead to fluid accumulation in the intestine and, hence, to diarrhoea. The observed increase in paracellular permeability could reflect disruption of tight junctions (TJs), which are important in the regulation of paracellular permeability in epithelial cells. TJs form the physical barrier to diffusion through the paracellular space, and the structure of these junctions may be indirectly regulated by the adjacent adherens junctions (AJs) [76]. TJs consist of assemblies of trans-membrane proteins, including claudins and occludin (an important sealing protein of TJs [77]), scaffolding proteins of the zona occludens family and signalling proteins, such as protein kinases [78] and protein phosphatases [79]. AJs in epithelial cells are formed by interaction between E-cadherin and catenins [80]. There is also evidence that peri-junctional actin [77] and myosin light chain [81] are involved in the regulation of the barrier function of TJs. 

Phosphorylation and dephosphorylation of components of the epithelial barrier have profound effects on paracellular permeability, and PP2A has been shown to play a crucial role in the formation and patency of tight junctions [82,83]. Formation of the E-cadherin/catenin complex is regulated by protein phosphatases [84], in this case, primarily by tyrosine phosphatase 1B [80]. The role of protein phosphatase inhibition and activation in the increase in paracellular permeability induced by OA and other protein phosphatase inhibitors therefore needs to be considered.

Phosphorylation of occludin plays a crucial role in the regulation of TJ integrity, and this protein undergoes dephosphorylation on Ser and Thr residues and hyperphosphorylation of Tyr residues during the disruption of TJs induced by various pharmacological and toxic substances, and enhanced PP2A activity, which induces dephosphorylation of occludin, is associated with increased paracellular permeability [79,85]. In this situation, therefore, inhibition of Ser/Thr phosphatases by OA would be expected to protect against increased paracellular permeability, rather than inducing such an effect. The increased paracellular permeability in Caco-2 cell monolayers induced by hydrogen peroxide and by acetaldehyde was indeed shown to be ameliorated by OA and/or fostriecin, another potent protein phosphatase inhibitor, or by knockdown of PP2A [79,86]. Under the conditions of the above experiments, therefore, OA-induced increases in paracellular permeability do not appear to be due to effects on occludin. Furthermore, OA and other protein phosphatase inhibitors or knockdown of PP2A or PP1 enhanced the integrity of tight junctions and accelerated the calcium-induced reassembly of tight junctions in Caco-2 cell monolayers [82]. OA also increased the calcium-induced biogenesis of TJs in Madin-Darby canine kidney (MDCK) cells through promotion of the phosphorylation and recruitment of proteins into TJs [83], and no increase in paracellular permeability was observed at OA concentrations of up to 250 nM. Such an effect was seen only at very high concentrations of OA, at which the cells became rounded, indicating gross cytotoxicity [83].

Hyperphosphorylation of myosin light chain (MLC) increases tight junction permeability [81]. Treatment of cultured human intestinal epithelial monolayers with OA at a concentration of 1 µM caused no increase in the phosphorylation of MLC [73], although higher levels (10 µM) did lead to an increase in the phosphorylation of this protein in MDCK cells [87].

Disruption of the F-actin cytoskeleton by OA has been observed in several cell types *in vitro* [58,74,88,89], and it was suggested that such a change could decrease the patency of TJs [74] and, thus, lead to diarrhoea *in vivo*. It has been shown, however, that methyl okadaate, which has little or no effect on the activities of PP2A and PP1, was of similar activity to OA in disrupting the F-actin cytoskeleton in hepatocytes [58] and in neuroblastoma cells [90]. It was suggested [58] that methyl okadaate may inhibit protein phosphatases other than PP1 and PP2A or that this compound and OA may have targets other than the protein phosphatases for their effects on the actin cytoskeleton. 

It has been suggested that effects on E-cadherin could contribute to the increased paracellular permeability induced by OA [91]. In one study [92], OA was shown to induce hyperphosphorylation of β-catenin exclusively on Ser/Thr residues in keratinocytes, although no effect of OA on the phosphorylation state of β-catenin was seen in human intestinal epithelial T_84_ monolayers [73]. OA has been reported to decrease the expression of E-cadherin in MCF-7 breast cancer cells [93], although the mechanism of this effect is presently unknown.

It has been concluded that the mechanisms underlying the ability of OA to enhance paracellular permeability are not fully understood [94]. There appear to be marked differences in the effect of OA on different cell types *in vitro*, and the evidence for the involvement of protein phosphatase inhibition in the induction of increased paracellular permeability is inconclusive.

Alternative pathways to increased paracellular permeability have been proposed. A proteomic study by Wang *et al.* [72] revealed that 58 proteins in the mouse intestine were altered in abundance after oral administration of OA. These proteins were identified as those involved in macromolecular metabolism, cytoskeleton reorganisation, signal transduction, molecular chaperoning and oxidative stress, and the authors concluded that multiple proteins, other than the protein phosphatases, could be involved in the diarrhoeagenicity of OA. OA induces TNFα release from cells *in vitro* [95], and this cytokine has been shown to increase paracellular permeability in cultured epithelial monolayers [96]. OA has also been shown to generate highly oxidising active oxygen species in cells *in vitro* [89,97,98], and oxidative stress is known to increase paracellular permeability [86,99]. Hosokawa *et al.* [75] demonstrated increased paracellular permeability in the rat colon *in vivo*, which was associated with the formation of micro-thrombi in the submucosal venules, followed by mucosal oedema. The change in paracellular permeability was attributed to increased permeability of the venule and subsequent mucosal damage. 

The epithelial barrier is known to be severely compromised when epithelial cells are lost, as occurs in intestinal erosion or ulceration [81]. Indeed, diarrhoea could simply result from the structural damage induced by OA in the intestine. Secretion of fluid occurs in the crypts, while absorption is mediated by the cells at the tips of the villi [100,101]. The crypts are largely undamaged by OA [20], so secretion will continue unabated, but loss of cells at the tips of villi will prevent fluid absorption, thus resulting in fluid accumulation in the intestinal lumen and, hence, diarrhoea. Reversible structural damage to intestinal epithelial cells, similar to that induced by OA, has been observed in animals following administration of irritant purgatives used in human medicine, such as castor oil [102,103], dioctyl sodium sulphosuccinate [104], bisacodyl [105], phenolphthalein [106] and sennosides and other anthranoid cathartics [107,108], none of which are known to inhibit protein phosphatases.

### 4.3. Protein Phosphatase Inhibition and Tumour Promotion by Okadaic Acid

The initial studies by Fujiki and co-workers on tumour promotion by OA and DTX-1 were followed by a series of experiments on the promoting activity of calyculin A, microcystin LR and nodularin, which, like OA, are potent inhibitors of protein phosphatases. Repeated application of calyculin A was as effective as OA in promoting DMBA-initiated skin carcinogenesis [109], while repeated intraperitoneal injections of microcystin LR or nodularin promoted tumour formation in the liver of rats following initiation with diethylnitrosamine [110,111]. These findings led to the conclusion that inhibition of protein phosphatases is a key factor in the mechanism of action of certain tumour promoters, classified as the “okadaic acid type promoters”, as distinct from promoters of the 12-*O*-tetradecanoylphorbol-13-acetate (TPA) type, and that promoting activity could be predicted on the basis of inhibitory effects on these enzymes.

In 1995, however, the Fujiki group reported on results with tautomycin, another inhibitor of PP1 and PP2A. Unlike the other protein phosphatase inhibitors, tautomycin had no effect on the incidence or multiplicity of skin tumours induced by DMBA, and this substance actually protected against cancer of the glandular stomach induced by MNNG, rather than increasing carcinogenic activity, as was expected. It was therefore concluded that inhibition of protein phosphatases is not a sufficient condition for tumour promotion [112].

Later studies showed the importance of irritant potential for tumour promotion in the skin of mice. OA, DTX-1 and calyculin A caused severe irritation when applied to the mouse ear, but tautomycin did not [25,26,109]. Similarly, oedema, inflammation and mucosal erosion were recorded in the stomach of mice after oral administration of OA or calyculin A, while no such effect was seen with tautomycin [22].

An ability to induce irritation and inflammation is a common property of tumour promoters [113,114,115], and the importance of irritation in tumour promotion is consistent with the suggestion [116,117] that pro-inflammatory cytokines, notably TNF-α, are of critical importance in this process. OA increased *TNF-α* gene expression in mouse skin and induced TNF-α release from cultured cells *in vitro* [95], as do many other irritant materials, including the “classical” tumour promoter TPA [117,118,119,120]. In contrast, tautomycin had no effect on cytokine production, and the inability of this substance to act as a tumour promoter was attributed to its failure to induce TNF-α [112]. It is possible that irritation and TNF-α induction are downstream effects of protein phosphatase inhibition, although this appears unlikely in view of tumour promotion by other irritant materials, such as TPA, lyngbyatoxin and palytoxin [121,122], which have not been reported to inhibit protein phosphatases. It is possible, therefore, that the tumour promoting ability of OA is simply due to its irritant potential, not to its ability to inhibit protein phosphatases.

### 4.4. Protein Phosphatase Inhibition and Neurotoxicity of Okadaic Acid

Changes in the cerebral activity of protein phosphatases have been implicated in the pathogenesis of Alzheimer’s disease. Individuals suffering from this disease suffer memory loss, and histological examination of the brains of such individuals has revealed neuronal neurodegeneration and the presence of neurofibrillary tangles within neurons and extracellular deposits of β-amyloid [123,124,125]. Neurofibrillary tangles result from accumulation of paired helical filaments within neurons, and such filaments consist largely of hyperphosphorylated tau protein [124]. Hyperphosphorylation of tau has been suggested to be caused by an increase in kinase activity or by a decrease in phosphatase activity within the neurons during the development of Alzheimer’s disease [126,127], and Arendt *et al.* [32] reasoned that injection of OA into the brain would increase tau phosphorylation by inhibiting PP2A, since this enzyme is predominantly responsible for the dephosphorylation of this protein [128]. Arendt *et al.* showed inhibition of PP1 and PP2A in the brain of rats following intracerebral injection [32,40,42], although others found no such effect [35]. As noted above, intracerebral injection of OA causes tau hyperphosphorylation, formation of neurofibrillary tangles and deposits of β-amyloid, together with memory loss and neurodegeneration. It was therefore suggested that intracerebral injection of OA, through its ability to inhibit protein phosphatases, would provide a useful model of Alzheimer’s disease [32].

There is evidence for and against this suggestion. The observation that calyculin A and microcystins, which, like OA, are protein phosphatase inhibitors, cause memory deficit and/or tau hyperphosphorylation and neuronal degeneration when injected into the rat brain [129,130,131] is evidence in support. However, DTX-1 did not induce neuronal death when injected into the hippocampus of rats [31], even though this substance is of similar potency to OA in inhibiting protein phosphatases. Furthermore, other cytotoxic compounds that are not known to inhibit protein phosphatases, such as kainic acid [132,133], domoic acid [134], isoproterenol [135] and the peroxynitrite generator, SIN-1 [136] cause similar changes after intracerebral injection. Furthermore, tau hyperphosphorylation is not dependent upon the administration of a protein phosphatase inhibitor, since this has been observed after injection of saline into the brain [32,137] and after transient cerebral ischaemia [138,139], and physical damage to the brain leads not only to hyperphosphorylation of tau, but also other cerebral changes characteristic of Alzheimer’s disease [140]. The weight of evidence is therefore against the hypothesis that the primary event in OA-induced cerebral toxicity involves protein phosphatase inhibition, and it has been argued that the observed changes may simply reflect a cellular response to neuronal injury [29,33,137].

## 5. Conclusion

Although it is widely accepted that the toxic effects of OA and derivatives are caused through inhibition of protein phosphatases, the evidence in support of such an association is very limited at the present time. In no case has a pathway from enzyme inhibition to toxic effect been identified, and the severity of the toxic effects of the OA derivatives are not in proportion to their inhibitory activity. Furthermore, the toxic effects induced by OA and derivatives are replicated by substances that are not protein phosphatase inhibitors, and known protein phosphatase inhibitors do not consistently exert the same toxic effects in animals as OA and derivatives. More work on the mechanism(s) of toxicity of these substances is required.
